# Inferring entire spiking activity from local field potentials

**DOI:** 10.1038/s41598-021-98021-9

**Published:** 2021-09-24

**Authors:** Nur Ahmadi, Timothy G. Constandinou, Christos-Savvas Bouganis

**Affiliations:** 1 Centre for Bio-Inspired Technology, Imperial College London, London, SW7 2AZ UK; 2 Department of Electrical and Electronic Engineering, Imperial College London, London, SW7 2AZ UK; 3 Care Research and Technology Centre, UK Dementia Research Institute at Imperial College London, London, UK; 4 School of Electrical Engineering and Informatics, Bandung Institute of Technology, Bandung, 40132 Indonesia; 5 Center for Artificial Intelligence (U-CoE AI-VLB), Bandung Institute of Technology, Bandung, 40132 Indonesia

**Keywords:** Neural decoding, Brain-machine interface

## Abstract

Extracellular recordings are typically analysed by separating them into two distinct signals: local field potentials (LFPs) and spikes. Previous studies have shown that spikes, in the form of single-unit activity (SUA) or multiunit activity (MUA), can be inferred solely from LFPs with moderately good accuracy. SUA and MUA are typically extracted via threshold-based technique which may not be reliable when the recordings exhibit a low signal-to-noise ratio (SNR). Another type of spiking activity, referred to as entire spiking activity (ESA), can be extracted by a threshold-less, fast, and automated technique and has led to better performance in several tasks. However, its relationship with the LFPs has not been investigated. In this study, we aim to address this issue by inferring ESA from LFPs intracortically recorded from the motor cortex area of three monkeys performing different tasks. Results from long-term recording sessions and across subjects revealed that ESA can be inferred from LFPs with good accuracy. On average, the inference performance of ESA was consistently and significantly higher than those of SUA and MUA. In addition, local motor potential (LMP) was found to be the most predictive feature. The overall results indicate that LFPs contain substantial information about spiking activity, particularly ESA. This could be useful for understanding LFP-spike relationship and for the development of LFP-based BMIs.

## Introduction

Extracellular recordings are one of the most widely used electrophysiological techniques and have been extensively used for basic neuroscience research (e.g. understanding neural coding and information processing) and clinical applications (e.g. brain-machine interface). The advent and advancement of microelectrode array technology have made it possible to simultaneously record neural signals from a large group of neurons^1–3^. The recorded raw neural signals are composed of two main components: local field potential (LFP) and spikes. LFPs are typically obtained by low pass filtering the raw neural signals (below $\sim$ 300 Hz) and are thought to mainly reflect summed synaptic activity from a local neuronal population (within a radius of at least a few hundred micrometres) around the recording electrode^4–8^. On the other hand, spikes are extracted by high-pass filtering the same raw neural signals (usually above $\sim$ 300 Hz) and subsequent spike processings. Based on these subsequent processings, spikes can be categorised into three types of signals, namely single-unit activity (SUA), multiunit activity (MUA) and entire spiking activity (ESA).

SUA is defined as the timing of spikes (i.e. action potentials) fired by individual neuron and is extracted via threshold crossing followed by unit classification known as spike sorting. MUA—sometimes also called multiunit spike (MSP)—refers to all the detected spikes (without spike sorting) and represents the aggregate spikes from an ensemble of neurons within a radius of $\sim$ 140–300 μm in the vicinity of the electrode tip^9–12^. Extracting both SUA and MUA relies on setting the threshold value (manually or automatically) which could be problematic when the recordings exhibit a low SNR or high variation over time. This circumstance is often encountered in chronic recordings where the amplitude of spikes decreases due to tissue responses and/or micromotion of the electrodes^13^. Moreover, threshold-based technique may result in a biased estimate of spiking activity in favour of large neurons (pyramidal neurons), hence leaving the spiking activity of small neuron undetected^14,15^.

Unlike both SUA and MUA which are represented by a sequence of binary signals, ESA is represented by a continuous signal and reflects an instantaneous measure of the number and size of spikes from a population of neurons around the recording electrode^16,17^. ESA is obtained through full-wave rectification (i.e. taking the absolute value) followed by low-pass filtering^15^. The term ESA is relatively new^18^ but its underlying principle has existed and been used for several decades^16,19^. Despite being less popular and less frequently used signal than its counterparts, ESA offers appealing advantages. Its threshold-less and automated processing provides a more reliable and less biased estimate of population spiking activity because it is less sensitive to random high-frequency noise and takes into account the spike contribution from small neurons. A number of studies have demonstrated that ESA can achieve better accuracy and reliability in measuring evoked responses (e.g. receptive field) in the visual cortex of monkeys while receiving various visual stimuli^14,15,20–22^. ESA has also been shown to yield more accurate and robust decoding of hand kinematics compared to SUA and MUA from three monkeys performing different tasks^23^.

Understanding the relationship between the LFP and spikes plays a critical role in addressing many issues in neuroscience research, such as neuronal functional organisation and connectivity, neuronal communication between different brain areas, cell assembly formation, neural coding and information processing^24–27^. In addition, it is also relevant for brain-machine interface (BMI), for example, measuring behavioural task-related information within different neural signals^28,29^ and extracting indirect spiking information features from LFPs in biofeedback based BMI^30^. As LFPs are thought to represent mainly the input to local neuronal networks, while spiking activity represents the output from local neuronal networks^31^, it is conceivable to relate the two signals based on system identification-based inference approach. Previous studies have shown that SUA^30,32,33^ and MUA^28,34^ can be inferred solely from LFPs with moderately good accuracy. So far, however, there has been no study that investigates the relationship between the LFPs and ESA.

In light of this, our present study aims to address the above-mentioned issue by using extracellular recordings from the motor cortex area of three macaque monkeys while performing two different tasks: point-to-point task and instructed delay reach-to-grasp task. We assess quantitatively how well ESA can be inferred from LFPs with multivariate multiple linear regression (MLR) method. We then analyse which features within LFPs are highly predictive of ESA. Furthermore, we compare the inference accuracy of ESA with those of SUA and MUA. We also investigate the impact of the number of LFP channels on the inference performance. Finally, we examine LFP channel importance for the inference performance.

## Results

### LFP feature informativeness for ESA inference

**Figure 1:**
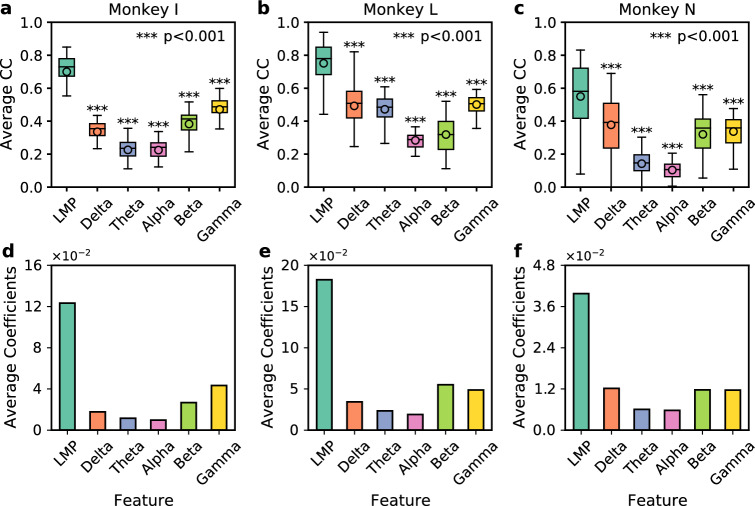
Comparison of ESA inference across different LFP features from three subjects. (**a**–**c**) Boxplot comparison of average CC across LFP features from monkey I, L, and N, respectively. Asterisks indicate LFP features whose inference performance differed significantly from that of LMP (***$p<0.001$). The horizontal lines and circles within the boxes indicate the median and mean, respectively. The boxes represent interquartile range (IQR) from 25th to 75th percentiles. The whisker extends 1.5 times the IQR. (**d**–**f**) Bar plot comparison of average coefficients (i.e. weights) across LFP features from monkey I, L, and N, respectively.

We evaluated and compared the informativeness of six different LFP features: the smoothed time-domain amplitude of LFP known as local motor potential (LMP) and average power spectra within five frequency bands (theta, delta, alpha, beta, and gamma). The informativeness of LFP features for the inference of ESA was measured using two approaches. First, we quantified and compared the inference performance of LFP features (in terms of average CC) with an MLR model that was fit independently (separately) on each LFP feature ($p=96$). Second, we took the absolute value of coefficients (weights) of an MLR model that was fit simultaneously on all LFP features combined ($p=576$). We then compared the average coefficients associated with each LFP feature. Figure 1a–c compare the average CC of six LFP features from monkey I, L, and N, respectively. Across 960 cases (10 blocks $\times$ 96 ESA channels) from each subject, LMP was found to consistently yield the highest inference performance (largest average CC) while power spectra in the alpha band (shortly referred to as alpha) consistently yielded the lowest inference performance (smallest average CC). LMP yielded an average CC of $0.70 \pm 0.02,$ $0.75 \pm 0.02,$ and 0.55 ± 0.02 for monkey I, L, and N, respectively [(mean ± standard error of the mean (SEM)]. The average CC of LMP was statistically significant different from that of other LFP features (***$p<0.001$). The order of informativeness of other LFP features (delta, theta, beta, and gamma) varied across subjects.

Consistent findings were also observed when using average coefficients as a metric for quantifying the informativeness of LFP features. LMP yielded the largest average coefficients (most informative) while alpha yielded the smallest average coefficients (least informative) across different subjects as shown in Fig. 1d–f. Similar to the case of average CC metric, the order of informativeness of other LFP features varied across subjects.

The results above were obtained using LFP signals filtered with low-pass filter at 100 Hz cut-off frequency. Next, we examined the impact of using higher cut-off frequency ($f_c=300$ Hz) that yields higher frequency component of gamma band ($30-300$ Hz). The performance comparison in terms of average CC and average coefficients obtained between using $f_c=100$ Hz and $f_c=300$ Hz is shown in Supplementary Figure 2 and is summarised in Supplementary Tables 3 and 4. We consistently found performance improvement of gamma with $f_c=300$ Hz compared to that of with $f_c=100$ Hz as shown in Supplementary Tables 3 and 4. The improvement in the case of average CC (average coefficient) metric was 4.26% (26.67%), 52% (32.65 %), and 50% (33.33%) for monkey I, L, and N, respectively. The performance improvement values from monkey I were quite different from that of monkey L and N. It is unclear what causes this difference. This could probably due to the fact that monkey I comes from a different dataset than the other two. When comparing the performance (absolute value) between LMP and gamma from a total of six cases (two metrics and three subjects), we found that LMP outperformed gamma in all six cases (100%) for $f_c=100$ Hz and five out of six cases (83.33%) for $f_c=300$ Hz. Since LMP was consistently found to be the most informative feature, we selected LMP as the feature for subsequent processing and analysis.

### Comparison of inference performance: ESA vs SUA vs MUA

**Figure 2:**
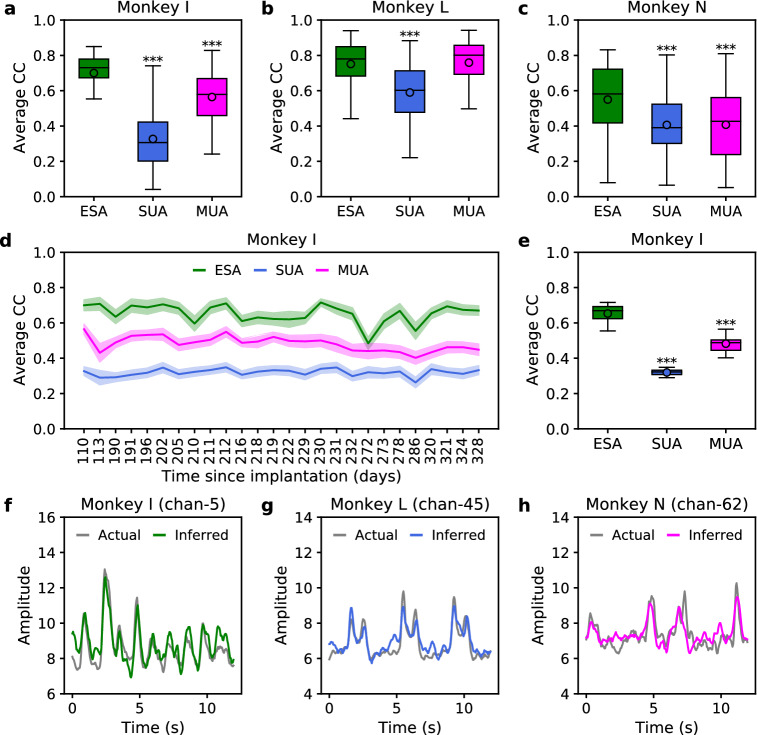
Comparison of inference performance in terms of average CC among different types of spiking activity across three subjects. (**a**–**c**) Boxplot comparison of average CC among ESA, SUA, and MUA from monkey I, L, and N, respectively. (**d**) Comparison of average CC among ESA, SUA, and MUA from monkey I over 26 recording sessions. (**e**) Boxplot comparison of average CC across 26 recording sessions. Asterisks indicate spiking activity whose inference performance differed significantly from that of ESA (***$p<0.001$). (**f**–**h**) A snippet example of actual and inferred ESA from monkey I (channel 5), monkey L (channel 45), and monkey N (channel 62), respectively.

We next compared the inference of ESA from LFP features to the inference of other types of spiking (SUA and MUA) from LFP features. We found the average of ESA as follows: $0.70 \pm 0.02,$ 0.75 ± 0.02, and $0.55 \pm 0.02$ for monkey I, L, and N, respectively. The average CC of SUA (MUA) were $0.33 \pm 0.01,$ $0.59 \pm 0.02,$ and $0.41 \pm 0.01$ ($0.56 \pm 0.02,$ $0.76 \pm 0.02,$ and 0.41 ± 0.02) for monkey I, L, and N, respectively. The average CC of ESA was statistically significantly larger than that of SUA (MUA) in three (two) subjects as can be seen from Fig. 2a–c. The higher inference performance of ESA was consistently observed across long-term recording sessions from monkey I as shown in Fig. 2d. The average CC of ESA, SUA, and MUA were $0.65 \pm 0.01,$ $0.32 \pm 0.01,$ and 0.48 ± 0.01, respectively. The relative inference performance of ESA was 2.03 and 1.35 times higher compared to those of SUA and MUA, respectively. Figure 2e displays boxplot of ESA inference that was statistically significant different than those of SUA and MUA (***$p<0.001$). Snippet examples of actual and inferred ESA from monkey I (channel 5), L (channel 45), and N (channel 62) are illustrated in Fig. 2f–h. Overall, the order of spiking activity from highest to lowest inference performance was ESA> MUA > SUA.

**Figure 3:**
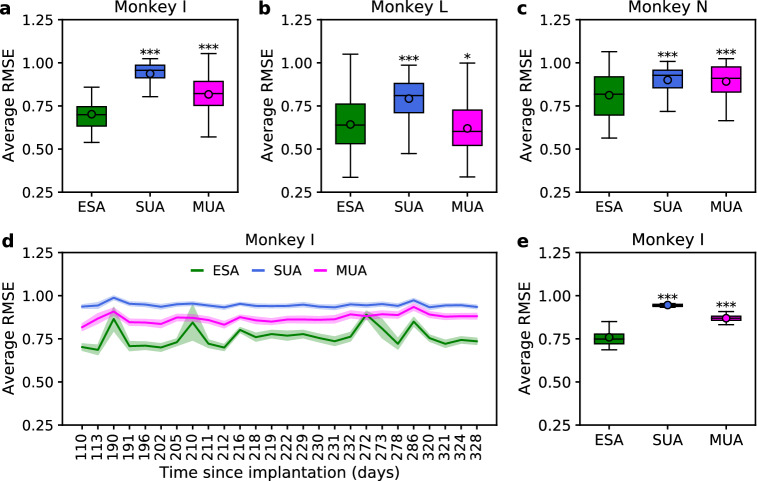
Comparison of inference performance in terms of average RMSE among different types of spiking activity across three subjects. (**a**–**c**) Boxplot comparison of average RMSE among ESA, SUA, and MUA from monkey I, L, and N, respectively. (**d**) Comparison of average RMSE among ESA, SUA, and MUA from monkey I over 26 recording sessions. (**e**) Boxplot comparison of average RMSE across 26 recording sessions. Asterisks indicate spiking activity whose inference performance differed significantly from that of ESA (*$p<0.05,$ ***$p<0.001$).

The above results were quantified in terms of average CC, which is a translation and scale invariant metric that only assesses the shape similarity between actual and inferred signals. In the presence of a large but constant inference error, the CC value can be high (implying falsely good accuracy). Therefore, as an additional metric for the inference performance, we also used RMSE that represents an average magnitude of inference error between actual and inferred signals. Similar to that of CC metric, the inference performance of ESA across three subjects and long-term recording sessions were consistently better (smaller RMSE) than those of SUA and MUA (see Fig. 3). The average RMSE of ESA, SUA, and MUA for monkey I were $0.70 \pm 0.01,$ $0.94 \pm 0.01,$ and $0.82 \pm 0.01,$ respectively. The average RMSE of ESA, SUA, and MUA for monkey L were $0.64 \pm 0.02,$ $0.79 \pm 0.01,$ and $0.62 \pm 0.02,$ respectively. The average RMSE of ESA, SUA, and MUA for monkey N were $0.81 \pm 0.01,$ $0.90 \pm 0.01,$ and $0.89 \pm 0.01,$ respectively. Figure 3d shows inference performance comparison among different types of spiking activity over long-term recording sessions. The average RMSE of ESA, SUA, and MUA were $0.76 \pm 0.01,$ $0.95 \pm 0.01,$ and 0.87 ± 0.01, respectively. We found statistically significant difference in average RMSE among ESA, SUA, and MUA (see Fig. 3e).

We were also interested in determining whether these findings could hold true when using different algorithms. We repeated the same procedures but using two different deep learning algorithms: multilayer perceptron (MLP) and long short-term memory (LSTM). More detailed description of MLP and LSTM used in this study along with their hyperparameter configurations can be found in Supplementary Information. Comparison of inference performance among ESA, SUA, and MUA using MLP and LSTM can be seen in Supplementary Figure 3 and Supplementary Figure 4, respectively. We observed similar trend where on average the inference performance of ESA was higher than those of SUA and MUA across subjects and long-term recording sessions. We then compared ESA inference performance under MLR, MLP, and LSTM algorithms. We found that the inference performance of MLR ($0.70 \pm 0.02,$ $0.75 \pm 0.02,$ and $0.55 \pm 0.02$) was lower than those of MLP ($0.73 \pm 0.02,$ $0.75 \pm 0.02,$ and 0.62 ± 0.02) and LSTM ($0.73 \pm 0.02,$ $0.76 \pm 0.01,$ and 0.63 ± 0.02) across three subjects (monkey I, L, and N, respectively). Relative to MLR, the deep learning algorithms yielded an average performance improvement of 4.15%, 0.53%, and 12.97% for monkey I, L, and N, respectively. There was statistically significant difference in inference performance among MLR, MLP, and LSTM within single recording session across three subjects. However, in the case of long-term recording sessions from monkey I, the difference in inference performance was not statistically significant as shown in Supplementary Figure 5.

### Impact of number of LFP channels on inference performance

**Figure 4:**
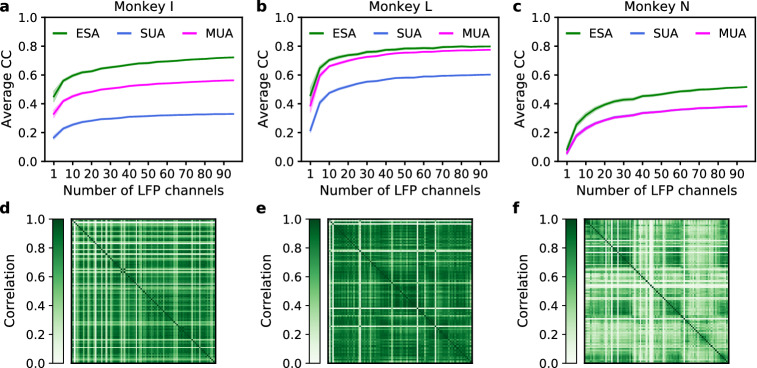
Impact of different number of LFP channels on inference performance (measured in average CC) across subjects. (**a**–**c**) Comparison of average CC among ESA, SUA, and MUA from monkey I, L, and N, respectively. The shaded areas represent 95% confidence intervals from 30 iterations. (**d**–**f**) Comparison of LFP interchannel correlation for monkey I, L, and N, respectively.

Next, we investigated the impact of different number of LFP channels on inference performance for different types of spiking activity. Performance comparison among ESA, SUA, and MUA across three subjects are plotted in Fig. 4a–c. The results across subjects showed that the inference performance of ESA was always higher than those of SUA and MUA regardless of the number of LFP channels. In addition, the inference performance of ESA, SUA, and MUA improved with the increasing number of LFP channels. Initially, the inference performance improved quickly but after a certain point, it only improved marginally (i.e. reaching plateau). However, the rate at which the inference performance reached a plateau was different among ESA, SUA, and MUA. Further analysis revealed that the inference performance of ESA reached a plateau quicker (in fewer number of LFP channels) than those of SUA and MUA. Averaging across three subjects, the inference performance of ESA, SUA, and MUA reached 90% of their maximum performance when using 35, 42, and 42 LFP channels, respectively.

Furthermore, we examined whether there is relationship between LFP interchannel correlation and the rate at which the inference performance reached a plateau. Comparison of LFP interchannel correlation across three subjects is plotted in Fig. 4d–f. The average LFP interchannel correlation for monkey I, L, and N were 0.63, 0.73, and 0.41, respectively. We found that the higher the LFP interchannel correlation, the faster the rate at which the inference performance of ESA, SUA, and MUA reached their plateau. The inference of ESA, SUA, and MUA reached their 90% of maximum performance when using 25, 30, and 35 LFP channels, respectively, for monkey I (10, 25, and 20 LFP channels, respectively, for monkey L; 70, 70, and 70 LFP channels, respectively, for monkey N).

### LFP channel importance for ESA inference

**Figure 5:**
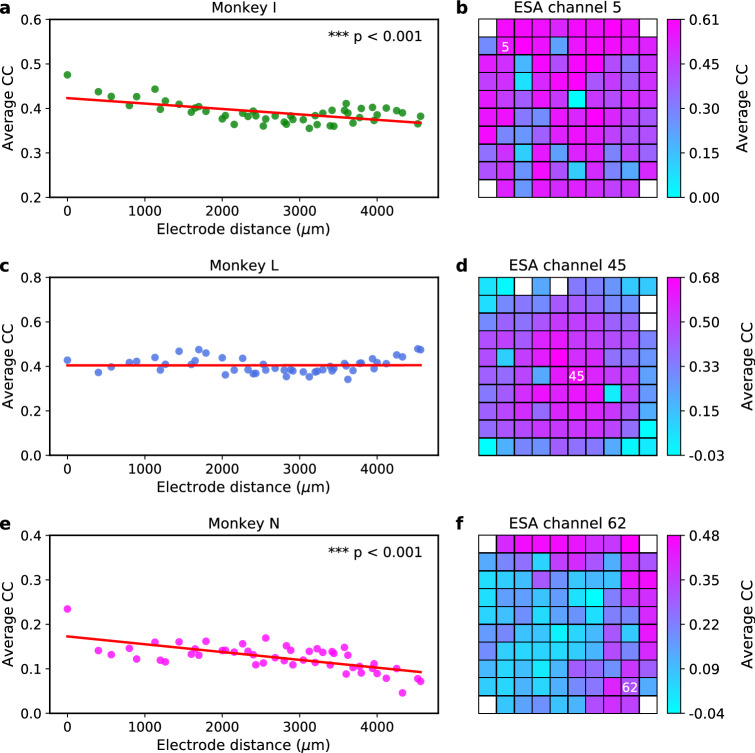
LFP channel importance score (quantified in terms of average CC) for ESA inference across subjects. (**a**,**c**,**e**) Scatter plot of LFP importance score over inter-electrode distance (μm) from monkey I, L, and N, respectively. Red solid lines represent linear regression lines used to test whether or not there is a significant linear trend between inter-electrode distance and LFP channel importance score. Asterisks indicate that there is a significant linear trend (two-tailed one-sample *t* test; **$p<0.01$, ***$p<0.001$). (**b**,**d**,**f**) Examples of heatmap of LFP channel importance score for ESA inference from monkey I (channel 5), monkey L (channel 45), and monkey N (channel 62), respectively. The importance score is mapped onto a $10 \times 10$ grid spatially corresponding to Utah electrode array configuration. white numbers inside the grids denote the ESA channel being inferred. White boxes on the grid represent unused (unconnected) electrodes. The larger the average CC, the more important is the channel for the inference.

Next, we assessed the LFP channel importance for the inference of all 96 ESA channels. The LFP channel importance was quantified using two metrics: average CC and average coefficient (see Methods section). It was then sorted based on the distance between all possible pairs of electrodes associated with the ESA channel output and LFP channel input. Figure 5a,c,e show scatter plots of average CC across interelectrode distances for monkey I, L, and N, respectively. We found statistically significant decreasing trends of average CC with the increase of interelectrode distance in two out of three subjects ($p<0.001$ for monkey I and N). Despite the decreasing trend, the shortest interelectrode distance did not necessarily yield the highest average CC. Similarly, the farthest the interelectrode distance also did not necessarily yield the lowest average CC. The average (maximum) CC for monkey I, L, and N was 0.40 (0.73), 0.38 (0.81), and 0.12 (0.66), respectively. Figure 5b,d,f show examples of LFP channel importance score (average CC) mapped onto 10-by-10 heatmap grids for ESA inference of channel 5 (monkey I), channel 45 (monkey L), and channel 62 (monkey N), respectively.

**Figure 6:**
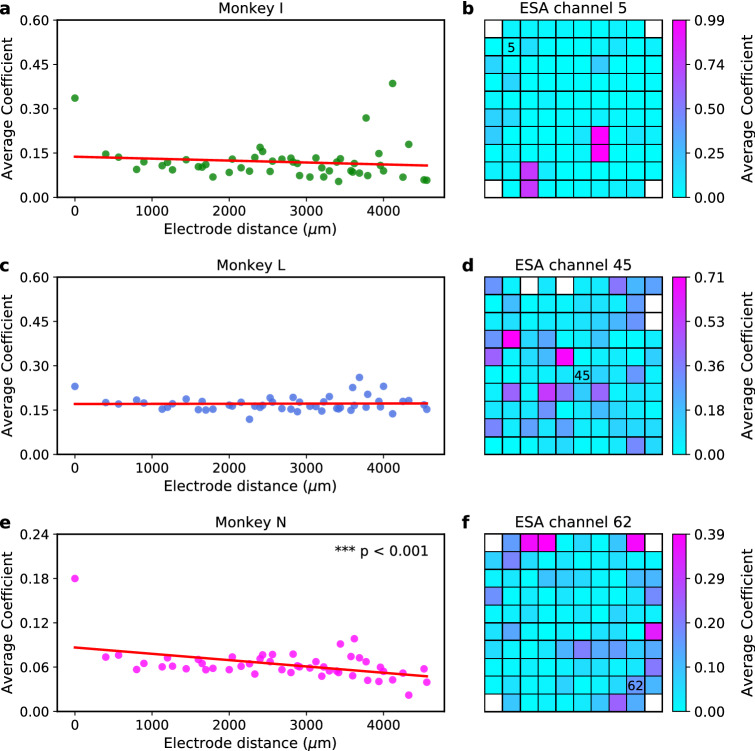
LFP channel importance score (quantified in terms of average coefficient) for ESA inference across subjects. (**a**,**c**,**e**) Scatter plot of LFP importance score over inter-electrode distance (μm) from monkey I, L, and N, respectively. Red solid lines represent linear regression lines used to test whether or not there is a significant linear trend between inter-electrode distance and LFP channel importance score. Asterisks indicate that there is a significant linear trend (two-tailed one-sample *t* test; ***$p<0.001$). (**b**,**d**,**f**) Examples of heatmap of LFP channel importance score for ESA inference from monkey I (channel 5), monkey L (channel 45), and monkey N (channel 62), respectively. The importance score is mapped onto a $10 \times 10$ grid spatially corresponding to Utah electrode array configuration. Black numbers inside the grids denote the ESA channel being inferred. White boxes on the grid represent unused (unconnected) electrodes. The larger the average coefficient, the more important is the channel for the inference.

When using average coefficient for quantifying LFP channel importance, we also observed decreasing trends (negative slope) in two out of three subjects. However, only one case (monkey N) was found to be statistically significant ($p<0.001$) as can be seen from Fig. 6a,c,e. Representative examples of LFP channel importance score for ESA inference of channel 5 (monkey I), channel 45 (monkey L), and channel 62 (monkey N) are plotted as heatmaps of $10 \times 10$ grid corresponding to the Utah array configuration, as shown in Fig. 6b,d,f.

Lastly, we also examined LFP channel importance score for the inference of SUA and MUA using the same approach as for ESA. Since each channel can have more than one SUA, we averaged the LFP channel importance score for SUA inference from the same channel. The results of LFP channel importance score for SUA inference using average CC and average coefficient metrics are visualised in Supplementary Figures 7 and 8, respectively. As for MUA inference, LFP channel importance score for MUA inference in terms of average CC and average coefficient are visualised in Supplementary Figures 9 and 10, respectively. From a total of six cases (3 subjects and two metrics), we found decreasing trends (negative slope) in four cases (three cases being statistically significant) for SUA and five cases (three cases being statistically significant) for MUA.

## Discussion

The present study investigates the relationship between local field potential (LFP) and entire spiking activity (ESA) by asking whether we can infer ESA solely from LFPs. In doing so, we firstly examined which feature within LFPs carries the highest information (i.e. most predictive) about ESA. Our experimental results revealed that LMP emerged as the most predictive feature. The power from high-frequency band (gamma) also showed high inference performance, especially when using 300 Hz cut-off frequency (see Supplementary Figure 2), although its inference performance was lower than that of LMP. On the other hand, the power from intermediate frequency bands carried only a little information about ESA. These findings were consistently observed across subjects performing different tasks. A considerable number of previous studies have reported that power modulation within high-frequency band ($>30\,$Hz) were highly informative, whereas the intermediate frequency bands were little informative about spiking activity (SUA and MUA)^26,32–35^. It is believed that the high-frequency band of LFPs are thought to reflect synchronised spiking activity arising from local neuronal population^32^. Our study confirms and extends the previous findings by showing that apart from power within high-frequency LFPs, LMP also contained substantial information about spiking activity. LMP even yielded the highest inference performance in both CC and RMSE metrics across subjects. Even though LMP has been frequently used as a feature for decoding behavioural tasks^36–38^, the use of LMP for inferring spiking activity has not been investigated. A rather similar feature to LMP is low-frequency LFP (lf-LFP) which is obtained by low-pass filtering the broadband LFPs^28,30^. The frequency band of lf-LFP is more clearly separated than that of LMP as low-pass filter results in better roll-off and stopband attenuation. On the other hand, LMP is obtained by a moving average filter which is simple, fast and yields less random white noise while maintaining the smoothing of LFP signals well^39^.

Given the highly informativeness of LMP, one may ask what the biophysical origin of LMP is. Unfortunately, the answer to this question remains to be established. It has been speculated that LMP is related to firing rate modulation of neuronal population near the recording electrode^40^ or distant neuronal population activity that is connected to the recording site^26,41^. It is also possible that LMP reflects evoked LFPs in the motor cortex in response to movement-related tasks^32,40^. A prior study suggested that LMP plays an important role not only in the execution but also in the preparation of movement (e.g. anticipation)^42^. This aligns well with our our results in which LMP showed the highest inference performance on movement experiment with and without preparatory delay interval (dataset II and I, respectively).

Next, we evaluated and compared the inference performance of ESA with those of SUA and MUA using LMP feature and multivariate multiple linear regression (MLR). Results across recording sessions and subjects showed that the inference performance of ESA was consistently and significantly higher than those of SUA and MUA. The same trends were also observed when using two deep learning based inference algorithms (MLP and LSTM). Previous studies have only conducted the inference of SUA and MUA from LFPs^28,30,32–34^. Therefore, to the best of our knowledge, our study is the first that evaluates the inference of ESA from LFPs and systematically compares its performance to those of SUA and MUA. LFP has been reported to have spatial reach within around 200–400 μm^21,43,44^ with other studies suggesting a broader spatial reach up to a few millimetres^45,46^. Our results may indicate that LFP, which has relatively broad spatial reach, is more related to population spiking activity than single-unit activity. ESA which reflects population spiking activity contains richer spiking information compared to SUA and MUA. Both SUA and MUA are typically obtained by using a threshold-based technique which is prone to false-positive or false-negative spike detection. If we set the threshold value too high, we could miss true but lower amplitude spikes; on the contrary, if we set the threshold too low, we may detect some background noises as spikes. In contrast to SUA and MUA, ESA does not use thresholding, instead, it employs full-rectification and low pass filter which are rather insensitive to random high-frequency noise and are able to preserve full information of spiking activity^15^. Moreover, thresholding favours large (pyramidal) neurons with high amplitude spikes and other neurons very close to the electrode tips^14,15^. The small neurons (with low amplitude spikes) or slightly farther neurons may not be detected. In contrast to that, ESA integrates the contribution from all neurons in the vicinity of the recording electrode tip.

We next investigated the impact of different number of LFP channels on the inference performance of spiking activity. The inference performance of ESA was found to be consistently higher than those of SUA and MUA regardless of the number of LFP channels. Moreover, ESA reached an inference performance plateau quicker (i.e. in fewer number of LFP channels) than SUA and MUA. This may suggest that ESA exhibits larger spatial coverage (encompassing smaller and farther neurons), which in turn contains more spiking information and higher interchannel correlation^23^. We also found that the higher LFP interchannel correlation (information redundancy), the quicker the inference performance of spiking activity reaches its plateau.

To gain more insights into the relationship between LFP and spiking activity, we quantified the LFP channel importance for ESA inference using two metrics, which are average CC and average coefficient. We sorted the average LFP channel importance scores according to the interelectrode distance in an ascending order. We found that four out of six cases (across three subjects and two metrics) exhibited decreasing linear trend (negative slope) between the LFP channel importance score and interelectrode distance with three cases being statistically significant. Similar trends were also observed in the case of SUA and MUA inferences. These results are in good agreement with the previous findings^26,34^ which showed that the inference performance degraded with the increase of interelectrode distance. This trend could be related to contribution of neuronal population giving rise to LFP signal which has been found to decay with the increasing electrode distance^5,7^. In addition, previous study reported that phase synchronisation between spiking activity and LFP (termed spike-field coherence) decreased with the increasing interelectrode distance^47^.

In this study, LFP was obtained using a low-pass filter with lower cut-off frequency (100 Hz) than the one typically used (300 Hz) to remove possible contamination by spike waveforms from nearby neurons^48^. Since slow modulation of spiking activity as low as 10 Hz can contribute to the LFPs^8,32,49^, this raised the question of how the cut-off frequency impacts the inference performance. We therefore performed the same procedure and compared the inference performance using different cut-off frequencies {10, 50, 100, and 300 Hz}. Results across subjects revealed that there was no significant difference in inference performance, indicating negligible contamination by nearby spike waveforms (see Supplementary Figure 6). This aligns well with previous studies which suggested that the contribution of slow modulation of spiking activity is negligible compared to other sources such as synaptic activity and membrane potential oscillations^24,50^.

Our study uses Utah array with fixed electrode depth (1 mm for dataset I and 1.5 mm for dataset II) which records neural signals from likely the same layer (presumably layer 5^51,52^). Since neural signals have been shown to exhibit different properties^53,54^ and contain different amount of task-related information^55,56^ across depths or layers, it is unclear whether the same findings can be observed using electrode with recording tips on varying depths (also known as laminar or linear electrode). This can be a potentially interesting avenue for future research.

In summary, we have shown that LFPs, in the form of LMPs, carry substantial information about spiking activity, particularly ESA. Since spiking activity has been widely used as an input signal for BMI, our finding corroborates the increasingly accumulating evidence that LFPs can be used as an alternative input signal. This is especially relevant for chronic recordings where the spike signals have been found to be unstable or even degrading over long periods of time. In this case, LFP-based BMI can be implemented using biomimetic approach for decoding behavioural parameters^38,57,58^. Alternatively, LFP can also be used to infer spiking activity which is then applied to BMI decoding using biofeedback (operant conditioning) approach^13,30^.

## Methods

### Neural recording and behavioural task

Electrophysiological recordings were obtained from two public neural datasets, herein referred to as dataset I^59^ and dataset II^60^. These datasets were recorded from the motor cortex area of three Rhesus macaque monkeys (*Macaca mulatta*) while performing predefined tasks with 96-channel silicon-based intracortical microelectrode (Utah) array. The data acquisition along with the behavioural task associated with each dataset are briefly described as follows.

#### Dataset I

This dataset was recorded from a male monkey (indicated by I) while performing a point-to-point task. The monkey had to to reach randomly drawn circular targets uniformly distributed around an 8-by-8 square grid. A sequence of new random targets was presented immediately and continuously after target acquisition without an inter-trial interval. The recordings were made by using a Utah array (platinum contact, 400 k$\Omega$ nominal impedance, 400 μm interelectrode spacing, 1 mm electrode length, and 4 mm $\times$ 4 mm area) referenced to a silver wire placed under the dura (several cm away from the electrodes). The recordings were pre-amplified and filtered with a 4th-order 7.5 kHz low-pass filter with a roll-off of 24 dB per octave. The recordings were then digitised with 16-bit resolution at 24.4 kHz sampling rate, which are hereinafter called as raw neural signals. More detailed description of the experimental setup is described elsewhere^61^. For our experiments and analyses, we used a total of 26 recording sessions spanning 7.3 months between the first (I20160627_01) and last (I20170131_02) sessions, with duration ranging from 6 to 13.6 min (average of $8.88 \pm 1.96$ min).

#### Dataset II

This dataset was recorded from two monkeys (female and male) indicated as L and N, respectively, while performing an instructed delayed reach-to-grasp task. The monkey had to grasp an object with one of grip types (side grip or precision grip) and displace it with either a high or low pulling force. In each trial, the monkey had to perform one of four possible trial types (from a combination of grip type and force type) randomly drawn from an equiprobable distribution. Before initiating the movement, the monkey had to wait for 1000 ms (preparatory delay). The trial was successful if the monkey could reach, grasp, pull and hold the object for 500 ms within the position window. The recordings were made by using a Utah array (iridium oxide contact, 50 k$\Omega$ average impedance at 1 kHz, 400 μm interelectrode spacing, 1.5 mm electrode length, and 4 mm $\times$ 4 mm area) referenced to two wires connected to the connector pedestal. The recordings were amplified and filtered with a 1st-order 0.3 Hz high-pass filter and a 3rd-order 7.5 kHz Butterworth low-pass filter. The recordings were then digitised with 16-bit resolution at 30 kHz sampling rate which are then called as raw neural signals. This dataset contains two recording sessions: L101210-001 (11.49 min) for monkey L and I140703-001 (16.43 min) for monkey N. A detailed description of the experimental setup and the corresponding behavioural task is provided elsewhere^62^.

### Signal processing and feature extraction

**Figure 7:**
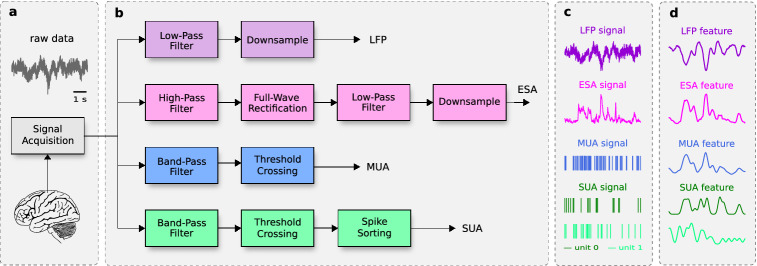
Schematic illustration of signal processing and feature extraction steps. (**a**) Raw neural signal acquisition from the motor cortex area of monkeys with a 96-channel intracortical Utah array. (**b**) Signal processing steps for different types of neural signals (LFP, ESA, MUA, and SUA). (**c**) LFP, ESA, MUA, and SUA signals obtained from the processing steps. (**d**) Extracted features from LFP, ESA, MUA, and SUA signals.

Signal processing steps from the raw neural signals to obtain LFP, ESA, MUA, and SUA along with their associated features are illustrated in Fig. 7a–d. We briefly describe the signal processing and feature extraction steps as follows.

#### Local field potential (LFP)

LFP was obtained by low-pass filtering the raw neural signal with a 4th-order Butterworth filter at 100 Hz and then downsampling it to 1 kHz. A cut-off frequency of 100 Hz was selected to eliminate possible contamination from multiunit spiking activity within higher LFP band ($100-300$ Hz). The filtering was performed in forward and backward directions to avoid any phase shift. We extracted six different LFP features consisting of average amplitude feature called local motor potential (LMP)^40^ and average spectral power features from five different frequency bands: delta (0.5–4 Hz), theta (4–8 Hz), alpha (8–12 Hz), beta (12–30 Hz) and gamma (30–100 Hz). The LMP was computed using time-domain moving average filter with 256 ms rectangular window. The spectral power feature was computed using short-time Fourier transform (STFT) with a 256 ms Hanning window and then averaged across frequency bins within each band. All the features were extracted through an overlapping fashion (206 ms overlap) to yield a sample every 50 ms.

#### Entire spiking activity (ESA)

ESA was obtained by high-pass filtering the raw neural signal with 1st-order Butterworth filter at 300 Hz. The filtered signal was then full-wave rectified, low-pass filtered with 1st-order Butterworth filter at 12 Hz and downsampled to 1 kHz. All the filtering processes were performed in both forward and backward directions. We extracted one ESA feature using time-domain moving average filter with a rectangular window of 256 ms width and 206 ms overlap (similar to that of LMP feature).

#### Multiunit activity (MUA)

Both datasets comprise the raw neural signals and pre-processed spikes (detected and sorted spikes). The spike waveforms were extracted by (i) filtering the raw neural signals with Butterworth filter (4th-order bandpass filter from 500 Hz to 5 kHz for monkey I; 4th-order high-pass filter at 250 Hz for monkey L; 2nd-order bandpass filter from 250 Hz to 5 kHz for monkey N), (ii) storing snippets of the filtered signals that crossed certain threshold values (48/64, 48, and 38 samples for monkey I, L, and N, respectively). MUA was defined as all the detected spikes and represented by their spike times. Details of the spike detection are described elsewhere by Makin et al.^61^ for dataset I and Brochier et al.^62^ for dataset II. We extracted spike rate feature by averaging the number of spikes within 256 ms window size (overlapped by 206 ms) to obtain feature sample every 50 ms. We only included MUA with spike rate exceeding 0.5 Hz for our experiments and analyses, which yielded 91, 96, and 96 units for monkey I, L, and N, respectively (see Table 1).

#### Single-unit activity (SUA)

SUA was obtained by aligning the extracted spike waveforms, reducing the dimensionality to a few principal components, and then sorting them into distinct putative single units via certain algorithms (operator defined templates for dataset I; K-Means and Valley Seeking for dataset II). Details of the spike sorting procedure for each dataset can be found in other studies^61,62^. We computed spike rate from SUA using the same method as in MUA. Only SUA with spike rate above 0.5 Hz were included in our experiments and analyses, yielding 157, 93, and 152 units for monkey I, L, and N, respectively (Table 1).

**Table 1:** Number of channels or units of neural signal types across subjects. For monkey I, the average number of units for MUA (SUA) over 26 recording sessions is $87.08 \pm 4.44$ (125.73 ± 13.95).

Subject	LFP	ESA	MUA	SUA
Monkey I	96	96	91	157
Monkey L	96	96	96	93
Monkey N	96	96	96	152

### Multivariate multiple linear regression (MLR)

MLR is a statistical technique to model multiple dependent (response) variables through a linear combination of multiple independent (predictor) variables. It is an extension of simple linear regression that uses only single response and predictor. MLR is mathematically formulated as follows:

1 $$\begin{aligned} y_{ik} = b_{0k} + \sum _{j=1}^{p} b_{jk}x_{ij} + e_{ik}, \quad {\text { for }} i \in \{1, \ldots , n \} {\text { and }} k \in \{1, \ldots , m \} \end{aligned}$$

where $y_{ik}$ is the *k*-th response for the *i*-th observation; $b_{0k}$ is the regression intercept for the *k*-th response; $b_{jk}$ is the *j*-th predictor’s regression slope (also called coefficient) for the *k*-th response; $x_{ij}$ is the *j*-th predictor for the *i*-th observation; $e_{ik}$ is multivariate Gaussian residual (error) term that accounts for all other factors influencing the response variables other than the predictors. Equation (1) can be written in matrix form as

2 $$\begin{aligned} {\mathbf {Y}} = {\mathbf {XB}} +{\mathbf{ E}} \end{aligned}$$

The regression coefficients (${\mathbf {B}}$) were estimated using ordinary least squares (OLS), that is, by minimising the sum of squared error (i.e. difference between the observed responses and predicted responses). The solution for OLS problem is given below.

3 $$\begin{aligned} {\hat {\mathbf{B}}} = {({\mathbf {X}}^{{\mathbf {T}}}{\mathbf {X}})^{-1}{\mathbf {X}}^{{\mathbf {T}}}{\mathbf {Y}}} \end{aligned}$$

where ${\hat {\mathbf{B}}}$ denotes the $(p+1) \times m$ coefficient matrix, ${\mathbf {X}}$ represents the $n \times (p+1)$ design matrix (LFP features), ${\mathbf {Y}}$ represents the $n \times m$ response matrix (ESA, SUA, or MUA). The MLR model was implemented using Scikit-learn^63^ (v0.22.1) machine learning library in Python programming language.

### Performance evaluation and metrics

Each dataset was divided into 10 non-overlapping contiguous blocks of equal size which were then categorised into three sets: training set (8 concatenated blocks), validation set (1 block) and testing set (1 block). We trained (fit) an MLR model on the training set and evaluated it on the testing set. We repeated the performance evaluation on 10 different blocks of testing set. The model’s input (LFP features) and output (ESA, SUA, or MUA features) were standardised (i.e. z-transformed) to have zero mean and unit variance. The model’s performance evaluation was assessed using Pearson’s correlation coefficients (CC) metric. CC measures the linear correlation between the actual and inferred spiking activity. In addition, we also evaluated the model performance using another metric called root mean square error (RMSE). It is a measure of the average magnitude of the inference error. Both CC^28,30,35,64,65^ and RMSE^28,35^ metrics have been used in several prior studies. CC and RMSE are defined as follows:

4 $$\begin{aligned} {\text {CC}} = \frac{\sum _{t=1}^{N}(y_t-\bar{y})(\hat{y}_t-\bar{\hat{y}}_t)}{\sqrt{\sum _{t=1}^{N}(y_t-\bar{y})^2}\sqrt{\sum _{t=1}^{N}(\hat{y}_t-\bar{\hat{y}}_t)^2}} \end{aligned}$$

5 $$\begin{aligned} {\text {RMSE}}=  \sqrt{ \sum _{i=1}^{N}(\hat{y}_i - y_i)^2/N} \end{aligned}$$

where $y_t$ and $\hat{y}_t$ represents the actual and inferred spiking activity at time step *t*, respectively and *N* is the total number of observations (i.e. samples).

### Impact of number of LFP channels on inference performance

To examine the impact of number of LFP channels on the inference performance, we selected randomly *p* distinct LFP channels where $p=\{1,5,10,15,\ldots ,90,95\}$ from a total of 96 channels. We then used these *p* LFP channels to train an MLR model and evaluate its performance for inferring all channels or units of spiking activity (ESA, SUA, and MUA). This procedure was repeated for 30 iterations to obtain the average inference performance along with its confidence interval. We also measured LFP interchannel correlation by computing Pearson’s correlation coefficient from all possible pair combination of LFP channels, yielding 96 $\times$ 96 correlation matrix.

### LFP channel importance for inference performance

The LFP channel importance for ESA inference performance was measured using two approaches. First, we quantified the LFP channel importance in terms of average CC with an MLR model that was fit independently (separately) on each LFP channel ($p=1$). Second, we took the absolute value of coefficients (weights) of an MLR model that was fit simultaneously on all LFP channels ($p=96$). We then averaged the coefficients associated with each LFP channel. The larger the average CC and average coefficients, the more important is that channel for ESA inference. We calculated the distance between the electrode location of ESA channels (output) and the electrode location of LFP channels (input). The LFP channel importance was then sorted based on the interelectrode distance in an ascending order. The interelectrode distance ranged from 0 to 4561 μm. We used the slope of linear regression to examine whether there is a significant linear trend between the interelectrode distance and LFP channel importance score (two-tailed one-sample *t* test).

### Statistical analysis

For each session, the mean and standard error of the mean (SEM) of the inference performance were evaluated across the number of units of spiking activity (ESA, SUA, and MUA) on 10 different blocks within the testing set. To test for significant effects between a pair of different LFP features or different spiking activity, we used a paired two-tailed *t*-test whenever the difference between the pairs follows normal distribution, otherwise we used Wilcoxon signed-rank test. The significance level ($\alpha$) was set to 0.05.

When the results are visualised using boxplot, the middle horizontal line and circle mark inside each box represent the median and mean, respectively. The coloured solid box represents interquartile range (IQR) from 25th to 75th percentiles. The whisker extends 1.5 times the IQR. All the analyses were conducted in Python (v3.6.10).

## Supplementary Information

Supplementary Information 1.

## Data Availability

Data are available from Zenodo at https://zenodo.org/record/583331 and from the German neuroinformatics node’s data infrastructure (GIN) at https://gin.g-node.org/INT/multielectrode_grasp.
