# Supplementary material for: Inferring entire spiking activity from local field potentials
Source: Sci Rep. 2021 Sep 24;11:19045. doi: 10.1038/s41598-021-98021-9 (PMC8463692; doi:10.1038/s41598-021-98021-9)
Supplement: Supplementary file 1 — Supplementary Information 1. [file 41598_2021_98021_MOESM1_ESM.pdf]

Inferring entire spiking activity from local field potentials

Nur Ahmadi[1,2,4,5*], **Timothy G. Constandinou**[1,2,3], **and Christos-Savvas Bouganis**[2]

[1]Centre for Bio-Inspired Technology, Imperial College London, London, SW7 2AZ, UK
[2]Department of Electrical and Electronic Engineering, Imperial College London, London, SW7 2AZ, UK
[3]Care Research & Technology Centre, UK Dementia Research Institute at Imperial College London
[4]School of Electrical Engineering and Informatics, Bandung Institute of Technology, Bandung, 40132, Indonesia
[5]Center for Artificial Intelligence (U-CoE AI-VLB), Bandung Institute of Technology, Bandung, 40132, Indonesia

SUPPLEMENTARY INFORMATION

Multilayer perceptron (MLP)

Multilayer perceptron (MLP) is a type of feedforward artificial neural network (ANN) that consists of at least three layers (an input layer, one or more hidden layers, and an output layer) of nodes. Each node is a neuron that produces an output by computing weighted sum of its inputs and passing through an activation function, which is formulated as

$$\mathbf{y} = \phi(\mathbf{W}\mathbf{x} + \mathbf{b}) \tag{1}$$

where $\mathbf{W}$ denotes the learnable parameters (weights), $\mathbf{x}$ represent the input vector, $\mathbf{b}$ is the bias vector, and ϕ is the activation function.

Except for the output layer which uses linear activation function ($\phi(z) = z$), we used a non-linear activation function called rectified linear unit (ReLU; $\phi(z) = \max(0, z)$). We implemented MLP model with a single hidden layer architecture. The number of units in the last (output) layer equals to the number of channels or units of spiking activity (see Table 1 in the paper). The MLP model was implemented using TensorFlow (v2.1.0) deep learning framework[1] and trained with Adam optimiser. Other hyperparameters including number of units (i.e. neurons), number of epochs, batch size, dropout rate and learning rate were determined through hyperparameter optimisation (described in the next section).

Long short-term memory (LSTM)

Long short-term memory (LSTM), proposed by Hochreiter and Schmidhuber in 1997[2], is one of the most popular deep learning architectures and has achieved state-of-the-art performance in a wide range of machine learning problems, especially those dealing with time-series data[3]. It addresses the problem of vanishing or exploding gradient commonly found in traditional recurrent neural networks (RNNs). LSTMs can effectively learn long-term temporal dependencies via a memory cell that maintains its state overtime and gating mechanism that controls the flow of information into and out of the memory cell. In this study, we employed a commonly used variant of LSTM architectures which consists of three gates (forget, input, output) and a single memory cell. The states of LSTM components at timestep t are formulated as:

$$\begin{aligned}
\mathbf{f}_t &= \sigma(\mathbf{W}_f\mathbf{x}_t + \mathbf{U}_f\mathbf{h}_{t-1} + \mathbf{b}_f) \\
\mathbf{i}_t &= \sigma(\mathbf{W}_i\mathbf{x}_t + \mathbf{U}_i\mathbf{h}_{t-1} + \mathbf{b}_i) \\
\tilde{\mathbf{c}}_t &= \tanh(\mathbf{W}_c\mathbf{x}_t + \mathbf{U}_c\mathbf{h}_{t-1} + \mathbf{b}_c) \\
\mathbf{o}_t &= \sigma(\mathbf{W}_o\mathbf{x}_t + \mathbf{U}_o\mathbf{h}_{t-1} + \mathbf{b}_o) \\
\mathbf{c}_t &= \mathbf{f}_t \odot \mathbf{c}_{t-1} + \mathbf{i}_t \odot \tilde{\mathbf{c}}_t \\
\mathbf{h}_t &= \mathbf{o}_t \odot \tanh(\mathbf{c}_t)
\end{aligned} \tag{2}$$

where $\mathbf{x}, \mathbf{h}, \mathbf{f}, \mathbf{i}, \mathbf{o}, \mathbf{c}$ consecutively represent the input, output, forget gate, input gate, output gate, and memory cell. σ and tanh denote the logistic sigmoid and hyperbolic tangent activation functions, respectively. The operator $\odot$ denotes the element-wise multiplication. Parameters that are learnt during training include input and recurrent weights ($\mathbf{W}, \mathbf{U}$) and bias vectors ($\mathbf{b}$).

We used 1-layer LSTM with number of timesteps (i.e. sequence length) empirically set to 3. The last timestep from the LSTM output was connected to a fully connected layer with number of units equals to the number of channels or units of spiking activity (see Table 1 in the paper). The LSTM model was implemented using TensorFlow (v2.1.0) and trained with RMSprop optimiser. Similar to that of the MLP model, other hyperparameters for LSTM model were selected via hyperparameter optimisation.

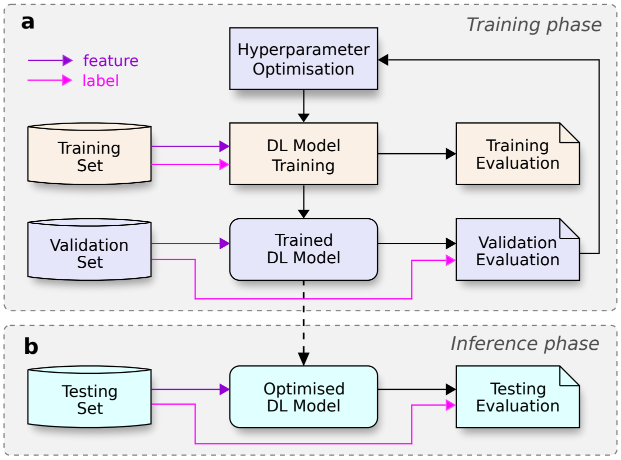

Suplementary Figure 1. Schematic illustration of training and inference phase of deep learning model. (**a**) Training and hyperparameter optimisation phase. (**b**) Inference (evaluation) phase.

Hyperparameter optimisation

Each dataset was divided into three sets: training, validation, and testing (same as described in Method section of the paper). The training set was used for model training, the validation set was used for hyperparameter optimisation, whereas the testing set was used for performance evaluation. The schematic of model training, optimisation and evaluation is illustrated in Supplementary Figure 1. We standardised (i.e. z-transformed) the input (LFP features) and output (ESA, SUA, or MUA features) to have zero mean and unit variance. The model was trained using root mean squared error (RMSE) loss function. We used Bayesian optimisation library called Optuna (v1.2.0)[4] to optimise the model's hyperparameter values from predefined hyperparameter value range (see Supplementary Tables 1 and 2) with 150 iterations. The hyperparameter optimisation was performed independently for each spiking activity (ESA, SUA, and MUA) and each subject (monkey I, L, and N) from LFP features (LMP). The resulting optimised hyperparameter values for MLP and LSTM models are listed in Supplementary Tables 1 and 2, respectively.

Hyperparameter	Value range	Optimised hyperparameter for MLP models								
		Monkey I			Monkey L			Monkey N		
		ESA	SUA	MUA	ESA	SUA	MUA	ESA	SUA	MUA
Number of units	$\{5, 10, \cdots, 150\}$	130	115	130	65	140	145	105	135	120
Number of epochs	$\{1, 2, \cdots, 25\}$	12	23	17	25	24	17	18	17	25
Batch size	$\{32, 64, 96\}$	96	96	64	64	32	32	96	32	96
Dropout rate	$\{0, 0.1, \cdots, 0.4\}$	0.2	0.4	0.4	0.1	0.4	0.2	0.3	0.4	0.4
Learning rate	$\{5, 10, \cdots, 100\} \times 10^{-4}$	0.0075	0.001	0.002	0.005	0.0015	0.0025	0.003	0.001	0.002

Suplementary Table 1. Hyperparameter configuration of LFP-based MLP models for inferring ESA, SUA, and MUA from 3 monkeys (I, L, and N). These hyperparameter values were optimised with LMP features as the input.

Hyperparameter	Value range	Optimised hyperparameter for LSTM models								
		Monkey I			Monkey L			Monkey N		
		ESA	SUA	MUA	ESA	SUA	MUA	ESA	SUA	MUA
Number of units	$\{5, 10, \cdots, 150\}$	135	130	125	140	105	125	150	105	135
Number of epochs	$\{1, 2, \cdots, 25\}$	23	12	16	19	19	24	19	17	20
Batch size	$\{32, 64, 96\}$	32	64	32	32	64	64	64	64	96
Dropout rate	$\{0, 0.1, \cdots, 0.4\}$	0.3	0.3	0.2	0.1	0.2	0.3	0.3	0.2	0.3
Learning rate	$\{5, 10, \cdots, 100\} \times 10^{-4}$	0.0015	0.001	0.001	0.0015	0.0025	0.003	0.001	0.001	0.0015

Suplementary Table 2. Hyperparameter configuration of LFP-based LSTM models for inferring ESA, SUA, and MUA from 3 monkeys (I, L, and N). These hyperparameter values were optimised with LMP features as the input.

LFP feature	Average CC								
	Monkey I			Monkey L			Monkey N		
	100 Hz	300 Hz	Diff (%)	100 Hz	300 Hz	Diff (%)	100 Hz	300 Hz	Diff (%)
LMP	0.70	0.70	0	0.75	0.75	0	0.55	0.55	0
Delta	0.34	0.34	0	0.49	0.49	0	0.38	0.38	0
Theta	0.23	0.23	0	0.47	0.47	0	0.14	0.14	0
Alpha	0.22	0.22	0	0.28	0.28	0	0.10	0.10	0
Beta	0.38	0.38	0	0.32	0.32	0	0.32	0.32	0
Gamma	0.47	0.49	4.26	0.50	0.76	52	0.34	0.51	50

Suplementary Table 3. Comparison of average CC across different LFP features from three subjects (monkey I, L, and N). Diff (%) represents percentage of difference when using cut-off frequency of 300 Hz compared to that of 100 Hz.

LFP feature	Average coefficients								
	Monkey I			Monkey L			Monkey N		
	100 Hz	300 Hz	Diff (%)	100 Hz	300 Hz	Diff (%)	100 Hz	300 Hz	Diff (%)
LMP	0.040	0.039	-2.50	0.183	0.155	-15.30	0.040	0.035	-12.50
Delta	0.006	0.006	0	0.034	0.031	-8.82	0.012	0.011	-8.33
Theta	0.003	0.003	0	0.023	0.021	-8.70	0.006	0.006	0
Alpha	0.003	0.003	0	0.019	0.017	-10.53	0.006	0.005	-16.67
Beta	0.009	0.008	-11.11	0.055	0.050	-9.09	0.012	0.011	-8.33
Gamma	0.015	0.019	26.67	0.049	0.065	32.65	0.012	0.016	33.33

Suplementary Table 4. Comparison of average coefficients across different LFP features from three subjects (monkey I, L, and N). Diff (%) represents percentage of difference when using cut-off frequency of 300 Hz compared to that of 100 Hz.

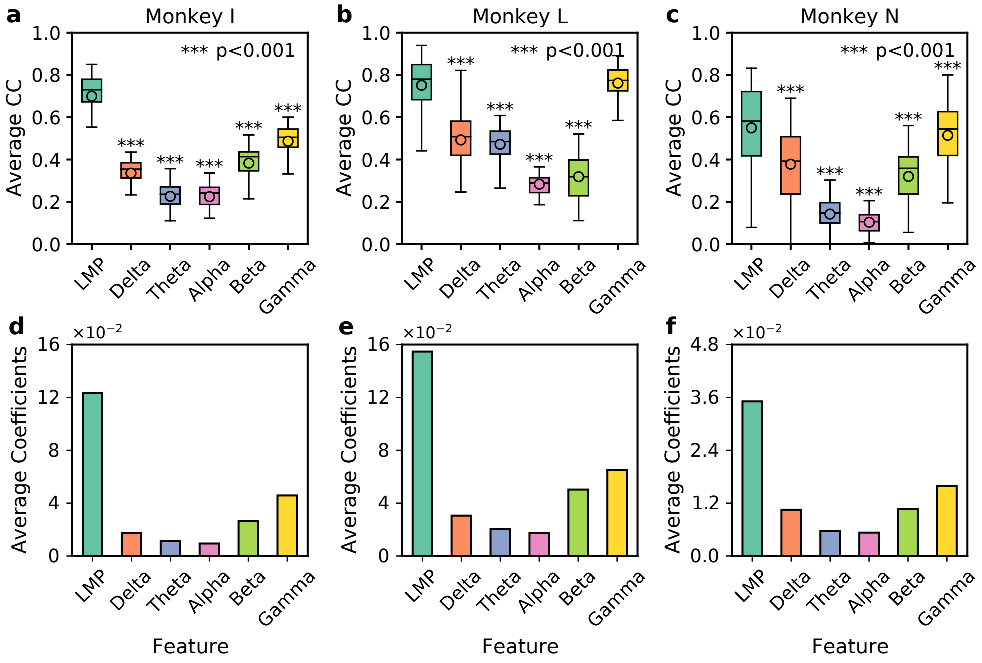

Suplementary Figure 2. Comparison of ESA inference across different LFP features from three subjects. The LFP signals were obtained by using low pass filter with 300 Hz cut-off frequency. (**a**–**c**) Boxplot comparison of average CC across LFP features from monkey I, L, and N, respectively. Asterisks indicate LFP features whose inference performance differed significantly from that of LMP (*** p<0.001). (**d**–**f**) Bar plot comparison of average coefficients (i.e. weights) across LFP features from monkey I, L, and N, respectively.

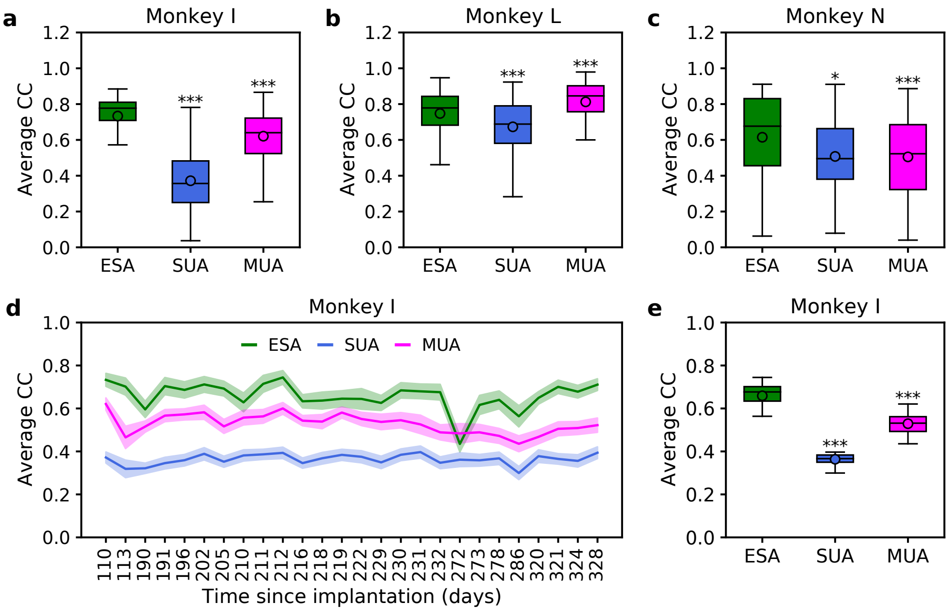

Suplementary Figure 3. Comparison of inference performance (in terms of average CC) using MLP among different types of spiking activity across three subjects. (**a**–**c**) Boxplot comparison of average CC among ESA, SUA, and MUA from monkey I, L, and N, respectively. (**d**) Comparison of average CC among ESA, SUA, and MUA from monkey I over 26 recording sessions. (**e**) Boxplot comparison of average CC across 26 recording sessions. Asterisks indicate spiking activity whose inference performance differed significantly from that of ESA (* p<0.05, *** p<0.001).

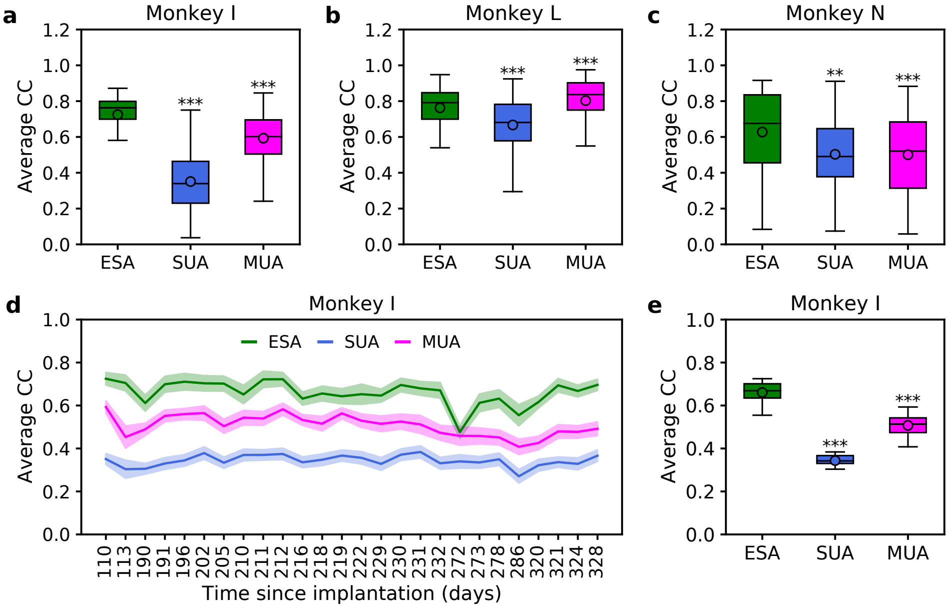

Suplementary Figure 4. Comparison of inference performance (in terms of average CC) using LSTM among different types of spiking activity across three subjects. (**a**–**c**) Boxplot comparison of average CC among ESA, SUA, and MUA from monkey I, L, and N, respectively. (**d**) Comparison of average CC among ESA, SUA, and MUA from monkey I over 26 recording sessions. (**e**) Boxplot comparison of average CC across 26 recording sessions. Asterisks indicate spiking activity whose inference performance differed significantly from that of ESA (** p<0.01, *** p<0.001).

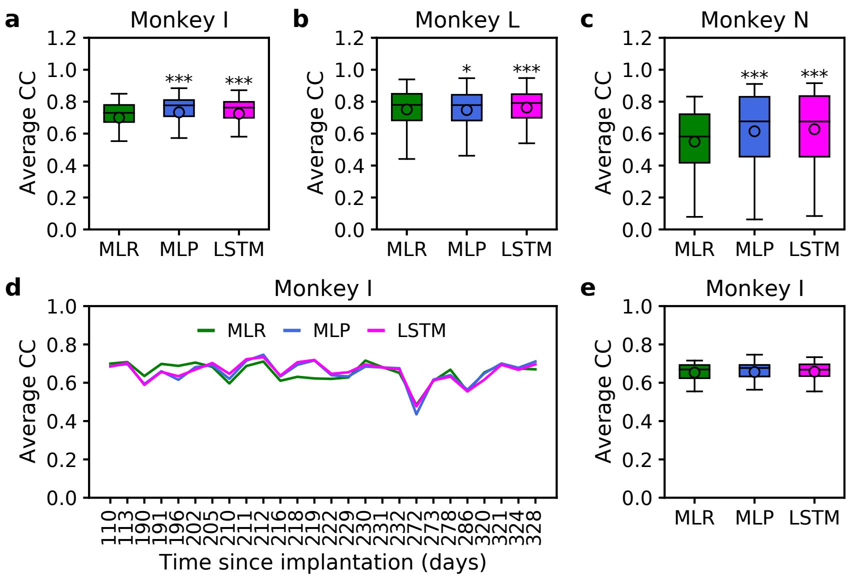

Suplementary Figure 5. Comparison of ESA inference performance (in terms of average CC) among different algorithms across three subjects. (**a**–**c**) Boxplot comparison of average CC among MLR, MLP, and LSTM from monkey I, L, and N, respectively. (**d**) Comparison of average CC among MLR, MLP, and LSTM from monkey I over 26 recording sessions. (**e**) Boxplot comparison of average CC across 26 recording sessions. Asterisks indicate algorithm whose inference performance differed significantly from that of MLR (* p<0.05, *** p<0.001).

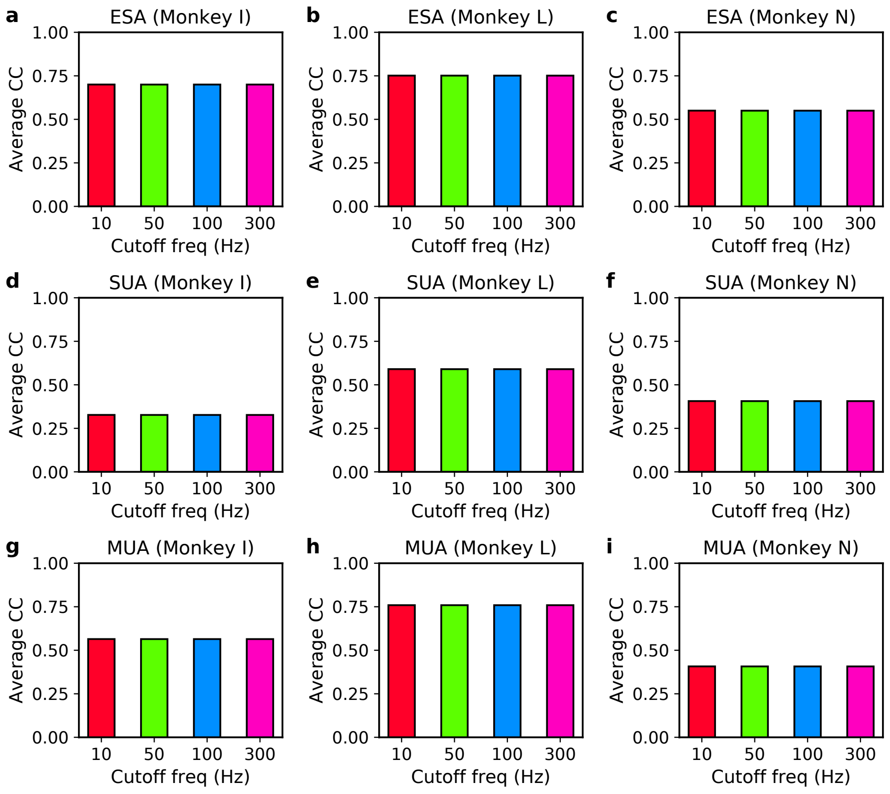

Suplementary Figure 6. Comparison of inference performance of spiking activity across different cut-off frequencies. (**a**–**c**) Comparison of ESA inference performance for monkey I, L, and N, respectively. (**d**–**f**) Comparison of SUA inference performance for monkey I, L, and N, respectively. (**g**–**i**) Comparison of MUA inference performance for monkey I, L, and N, respectively.

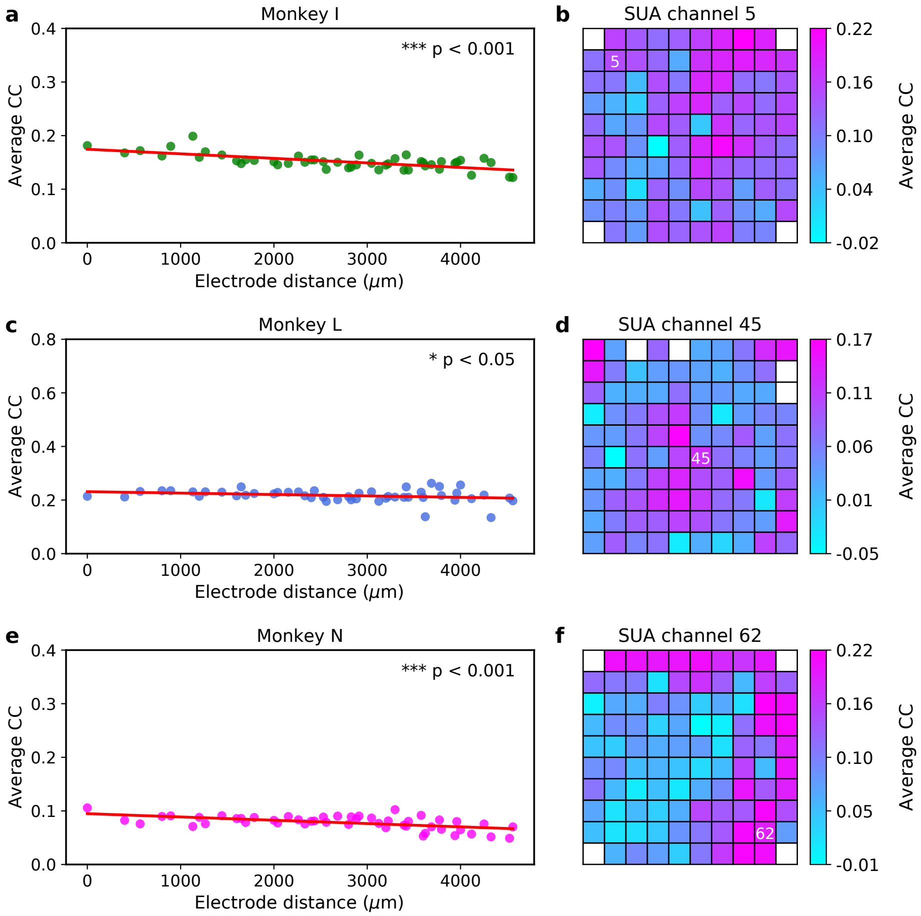

Suplementary Figure 7. LFP channel importance score (quantified in terms of average CC) for SUA inference across subjects. (**a**,**c**,**e**) Scatter plot of LFP importance score over inter-electrode distance (μm) from monkey I, L, and N, respectively. Red solid lines represent linear regression lines used to test whether or not there is a significant linear trend between inter-electrode distance and LFP channel importance score. Asterisks indicate that there is a significant linear trend (two-tailed one-sample t-test; * $p<0.05$, *** $p<0.001$). (**b**,**d**,**f**) Examples of heatmap of LFP channel importance score for SUA inference from monkey I (channel 5), monkey L (channel 45), and monkey N (channel 62), respectively. The importance score is mapped onto a 10×10 grid spatially corresponding to Utah electrode array configuration. White numbers inside the grids denote the SUA channel being inferred. White boxes on the grid represent unused (unconnected) electrodes. The larger the average CC, the more important is the channel for the inference.

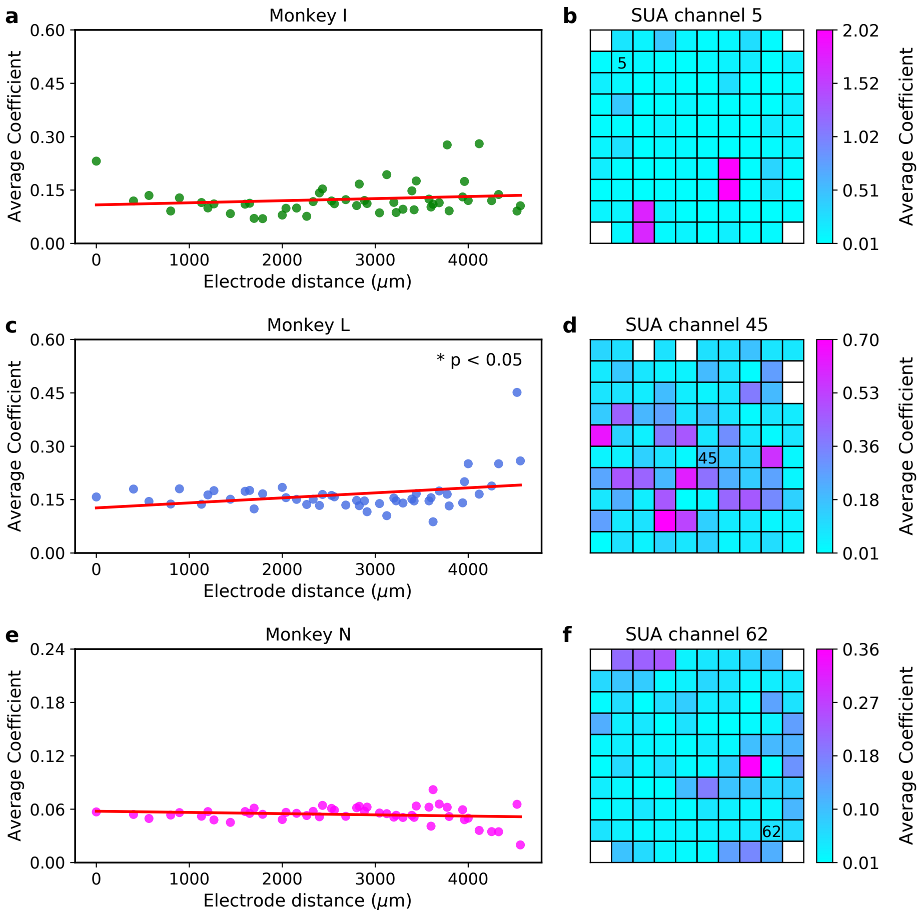

Suplementary Figure 8. LFP channel importance score (quantified in terms of average coefficient) for SUA inference across subjects. (**a**,**c**,**e**) Scatter plot of LFP importance score over inter-electrode distance (μm) from monkey I, L, and N, respectively. Red solid lines represent linear regression lines used to test whether or not there is a significant linear trend between inter-electrode distance and LFP channel importance score. Asterisks indicate that there is a significant linear trend (two-tailed one-sample t-test; * p<0.05). (**b**,**d**,**f**) Examples of heatmap of LFP channel importance score for SUA inference from monkey I (channel 5), monkey L (channel 45), and monkey N (channel 62), respectively. The importance score is mapped onto a 10×10 grid spatially corresponding to Utah electrode array configuration. Black numbers inside the grids denote the SUA channel being inferred. White boxes on the grid represent unused (unconnected) electrodes. The larger the average coefficient, the more important is the channel for the inference.

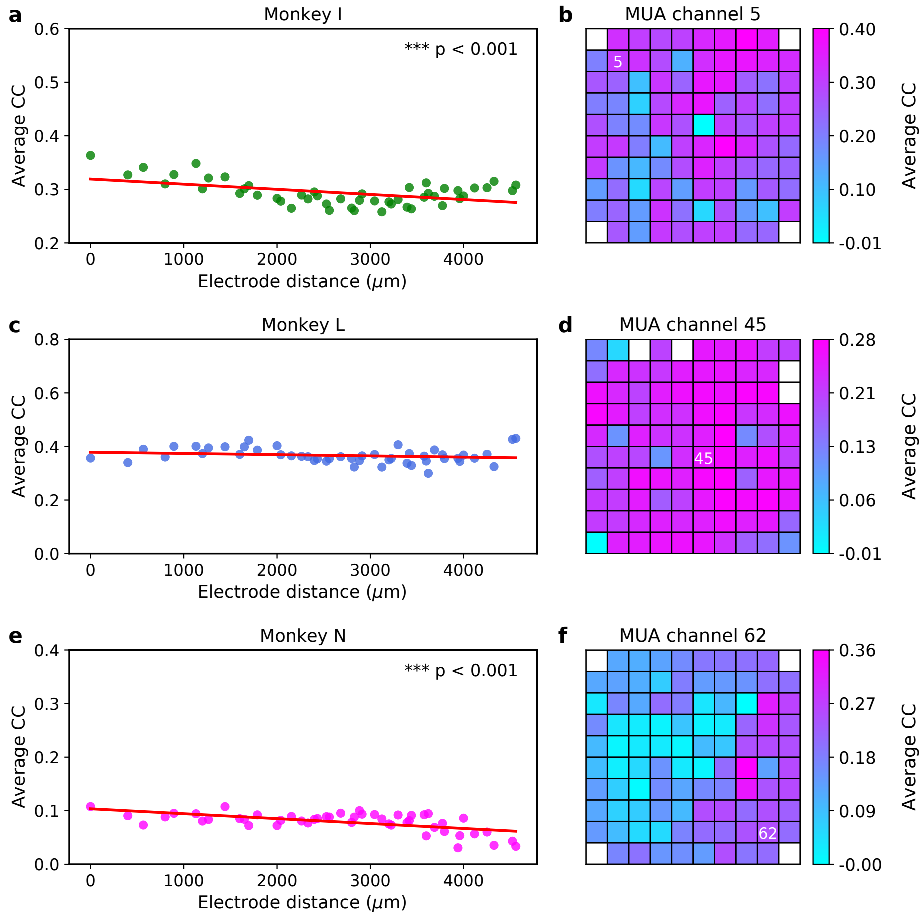

Suplementary Figure 9. LFP channel importance score (quantified in terms of average CC) for MUA inference across subjects. (**a**,**c**,**e**) Scatter plot of LFP importance score over inter-electrode distance (μm) from monkey I, L, and N, respectively. Red solid lines represent linear regression lines used to test whether or not there is a significant linear trend between inter-electrode distance and LFP channel importance score. Asterisks indicate that there is a significant linear trend (two-tailed one-sample t-test; * p<0.05). (**b**,**d**,**f**) Examples of heatmap of LFP channel importance score for MUA inference from monkey I (channel 5), monkey L (channel 45), and monkey N (channel 62), respectively. The importance score is mapped onto a 10$\times$10 grid spatially corresponding to Utah electrode array configuration. White numbers inside the grids denote the MUA channel being inferred. White boxes on the grid represent unused (unconnected) electrodes. The larger the average CC, the more important is the channel for the inference.

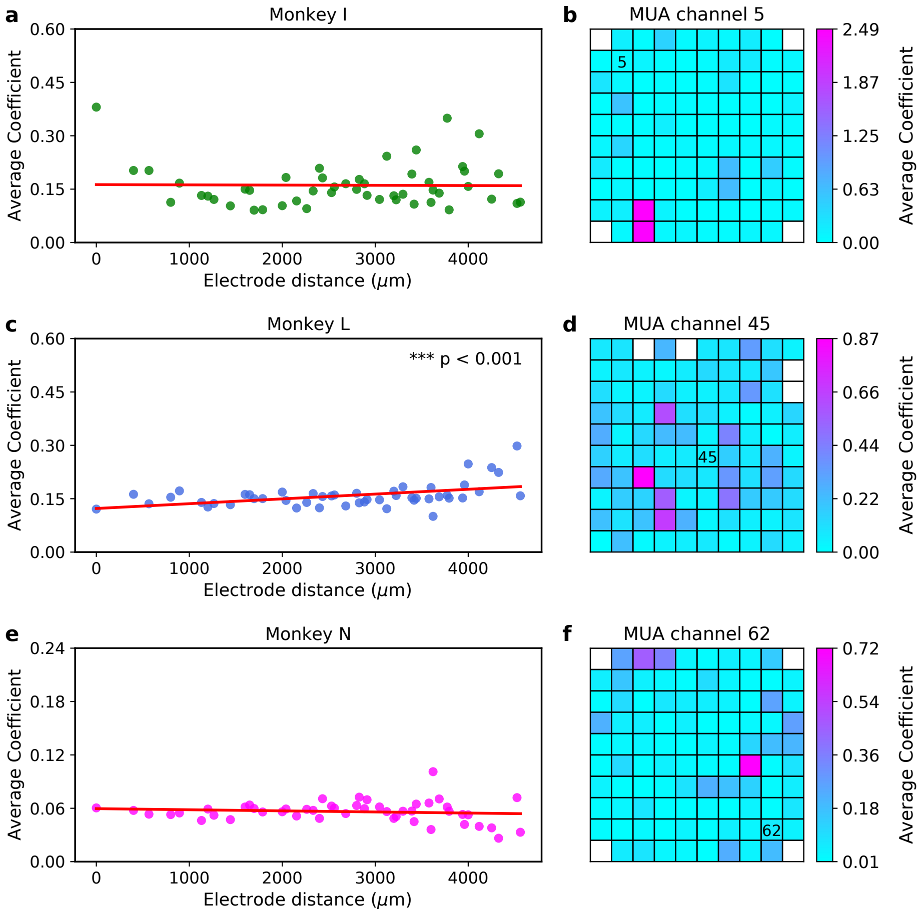

Suplementary Figure 10. LFP channel importance score (quantified in terms of average coefficient) for MUA inference across subjects. (**a**,**c**,**e**) Scatter plot of LFP importance score over inter-electrode distance (μm) from monkey I, L, and N, respectively. Red solid lines represent linear regression lines used to test whether or not there is a significant linear trend between inter-electrode distance and LFP channel importance score. Asterisks indicate that there is a significant linear trend (two-tailed one-sample t-test; *** p<0.001). (**b**,**d**,**f**) Examples of heatmap of LFP channel importance score for MUA inference from monkey I (channel 5), monkey L (channel 45), and monkey N (channel 62), respectively. The importance score is mapped onto a 10×10 grid spatially corresponding to Utah electrode array configuration. Black numbers inside the grids denote the MUA channel being inferred. White boxes on the grid represent unused (unconnected) electrodes. The larger the average coefficient, the more important is the channel for the inference.

References

1. Abadi, M. *et al.* Tensorflow: A system for large-scale machine learning. In *Proc. Symp. OS Des. Implement. (OSDI)*, 265–283 (2016).
2. Hochreiter, S. & Schmidhuber, J. Long short-term memory. *Neural Comput.* **9**, 1735–1780, DOI: 10.1162/neco.1997.9.8.1735 (1997).
3. Greff, K., Srivastava, R. K., Koutník, J., Steunebrink, B. R. & Schmidhuber, J. LSTM: A search space odyssey. *IEEE Trans. Neural Netw. Learn. Syst.* **28**, 2222–2232, DOI: 10.1109/TNNLS.2016.2582924 (2016).
4. Akiba, T., Sano, S., Yanase, T., Ohta, T. & Koyama, M. Optuna: A next-generation hyperparameter optimization framework. In *ACM Proc. Int. Conf. Knowl. Discov. Data Min. (KDD)*, 2623–2631, DOI: 10.1145/3292500.3330701 (2019).
